# Thermal Regulation Performance of Shape-Stabilized-Phase-Change-Material-Based Prefabricated Wall for Green Grain Storage

**DOI:** 10.3390/ma16030964

**Published:** 2023-01-20

**Authors:** Changnv Zeng, Chaoxin Hu, Wanwan Li

**Affiliations:** School of Civil Engineering, Henan University of Technology, Zhengzhou 450001, China

**Keywords:** prefabricated SSPCM granary wall, green grain storage, heat transfer characteristics, thermal conductivity

## Abstract

In order to meet the great demand for green grain storage and low carbon emissions, paraffin, high-density polyethylene (HDPE), and expanded graphite (EG) were used to produce shape-stabilized phase change material (SSPCM) plates, which were then used to reconstruct building walls for existing granaries. A new type of SSPCM plate was then prefabricated with different thermal conductivities and a high latent heat. This plate could be directly adhered to the existing granary walls. In order to evaluate the thermal regulation performance of these phase change granary walls, experiments and numerical methods were established, specifically for the summer condition. The thermal behavior of the SSPCM granary wall was compared with that of the common concrete granary wall to obtain the optimal parameters. It was concluded that increasing the thickness of the SSPCM layer can reduce the temperature rise of the wall. However, the maximum latent heat utilization rate and energy storage effects were obtained when the SSPCM thickness was at an intermediate level of 30 mm. The thermal conductivity of the SSPCM had a controversial effect on the thermal resistance and latent heat utilization behaviors of the SSPCM. Considering the temperature level and energy saving rate, a 30 mm thick SSPCM plate with a thermal conductivity of 0.2 W/m·K provided a superior performance. When compared to the common wall, the optimized energy-saving rate was greatly enhanced by 35.83% for the SSPCM granary wall with a thickness of 30 mm and a thermal conductivity of 0.2 W/m·K.

## 1. Introduction

The green granary uses green, environmental-protection technology to ensure the quality and safety of grain storage, importantly to avoid the pollution of the grains and the environment [1]. The granary temperature is affected by the outdoor ambient air temperature and solar radiation. For most old granaries, the poor thermal insulation performance of the envelope enclosure easily leads to the penetration of substantial ambient heat into granary space. This will cause the grain temperature to exceed 40 °C [2]. A further consequence is that grain mildew and insect pests are induced. In order to provide a safe temperature for stored grain, most old granaries use an air-conditioning system to maintain a low temperature condition. Alternatively, old granaries use pesticides to avoid insect pests. These will cause a high electric energy consumption or chemical pollution to the grain and the environment. Regardless, high-quality grain cannot be provided, especially during long-term grain storage in China. Therefore, it is of great importance to transform these old granaries into green granaries to ensure energy saving and the environmental protection of grain storage.

The addition of thermal insulation boards to improve the thermal resistance of the building envelope has been commonly mostly used in the renovation of old granaries. Derradji et al. [3] used an expanded polystyrene insulation board to enhance the internal insulation of the building wall to control the indoor temperature. Different kinds of insulation materials, such as extruded polystyrene, mineral wool, polyurethane, etc., were applied to the granary roof [4]. The addition of thermal insulation layers increased the thermal resistance of the granary envelope and effectively reduced the granary temperature, which was influenced by the environment temperature. However, the existing renovations, performed by adding different thermal insulation materials to the old granaries, are high-energy consumption processes. For example, it is easy for the insulation to become damaged, fall off, and reduce performance when laid on the wall. Most importantly, the existing methods cannot regulate the self-induced grain temperature, especially in the large-capacity granaries. Thus, extra air-conditioning is usually used to regulate the grain temperature during long-term storage; this also leads to high energy consumption. Therefore, it is of great importance to develop new granary-renovation technologies for low energy consumption.

Phase change materials (PCMs), called energy-saving and self-temperature-regulated materials, can be integrated into the building envelope. PCMs can be used for different climates or seasons in different countries to manage the thermal environment and save energy with low energy and decreased carbon emissions [5]. They can store and release a large amount of latent heat during their phase change process, which makes them maintain a relatively constant temperature when temperature ranges among the phase change temperature of PCMs. Thus, reconstructed granary walls utilizing PCMs will enable granaries to have a high energy-storage capacity, effectively improving the walls’ temperature-controlling and heat-insulating performance [6]. The PCM granary wall may provide a promising green technology for the wall itself.

Many relevant theories and practices regarding PCMs are used in civil buildings to maintain a comfortable indoor environment [7,8]. For example, PCM particles were mixed into cement mortar and concrete to enhance their heat-storage capacity for regulating the indoor temperature of buildings [9,10,11]. However, the latent heat of the phase change mortar and concrete were lower than that of the PCM, which restricted its regulation ability on reducing the indoor temperature [12].

Although relevant theories of PCMs in civil buildings can be applied to the granary wall, granaries need to be controlled to a lower temperature. For example, the grain temperature needs to be below 25 °C to decrease insect harm to the stored grain and to maintain a stable temperature for long-term storage. Primarily, the interior temperature is specified to be no more than 25 °C for the quasi-low temperature granary [13], while the interior temperature should be for comfortable civil buildings 28 °C [14]. This causes different demands of the phase change temperature and heat-storage capacity.

More importantly, the grain storage industry focuses on the energy-saving reconstruction of existing granary buildings rather than launching new construction, which is quite different from civil buildings [15]. This leads to the conclusion that prefabricated wall construction technology should be used in the granary. However, the existing phase change walls in civil buildings are usually constructed by mixing phase change particles with common building materials, such as mortar or concrete, which is inapplicable to prefabricated walls. Given that, in previous studies, the PCM plate could be applied to the light walls to increase their thermal inertia and adjust the indoor temperature of buildings [16,17], we propose a prefabricated, shape-stabilized PCM (SSPCM) plate with a high latent heat integrated into the granary wall.

Moreover, a common problem is that the high cost of PCM for a low phase change temperature was unsuitable for large-scale applications in civil buildings and granaries.

To deal with the above problems, a prefabricated SSPCM plate with a lower phase change temperature and a lower cost was prepared for direct use in the granary wall. Paraffin is one of the most popular PCMs because of its high thermal stability, high latent heat capacity, consistent phase-transition temperature, low price, availability without flammability, toxic distortion, and crystallization problems [18]. However, paraffin leaks easily during the melting process, and its thermal conductivity is relatively low: approximately 0.2 W·m^−1^ K^−1^ [19]. To deal with the leakage problem of paraffin, an HDPE with a similar straight-molecular-chain structure to paraffin was used as a supporting material to wrap the paraffin, which can greatly reduce the melting leakage of paraffin [20]. Additionally, the high tenacity of HDPE may enhance the mechanical strength and structural stability of the SSPCM.

In order to enhance the thermal conductivity, EG was added into the mixture of paraffin and HDPE. It may improve the phase change rate and the utilization of the whole phase change latent capacity [21].

Therefore, a new type of prefabricated SSPCM plate with paraffin, HDPE, and EG was prepared for the granary wall in this study. Based on the prefabricated SSPCM plate, the experimental and physical models were established to investigate the thermal performance of the SSPCM granary wall. Its heat transfer behavior was then tested and simulated to select the optimal parameters of the SSPCMs. Furthermore, taking the common granary wall as a reference, the energy-saving effect of the SSPCM granary wall was evaluated.

## 2. Materials and Method

### 2.1. Experimental Section

#### 2.1.1. Preparation of SSPCM Plate

**Figure 1:**
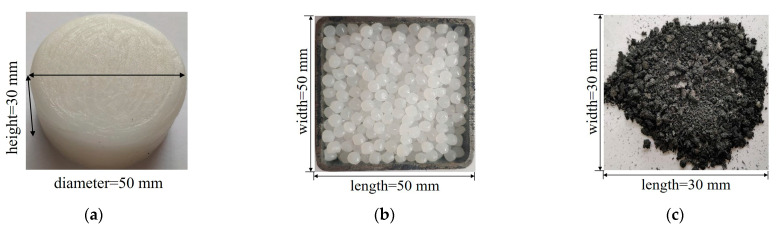
The raw materials of SSPCM: (**a**) solid–liquid mixed paraffin, (**b**) HDPE particle, (**c**) expanded graphite powder.

In this paper, the SSPCM was prepared with paraffin, high-density polyethylene (HDPE), and expanded graphite (EG), shown in in Figure 1. Considering that mixing paraffin has a much lower cost than the existing low-temperature pure paraffin, the solid–liquid paraffin mixture was selected for the phase change matrix, as is shown in Figure 1a. The mixture was prepared using solid and liquid paraffin with a mass ratio of 7:3. The phase change temperature and latent heat of solid paraffin were 52 °C and 252.86 J/g, respectively, while the pour point of liquid paraffin was −10 °C [22]. The phase change temperature and the latent heat of mixed paraffin were then 28.7 °C and 181.6 J/g, respectively. Due to its high compatibility with the paraffin mixture, HDPE was used as the supporting material. It had a a melting point of 160 °C, as is shown in Figure 1b. The EG was selected as a thermal-conductivity enhancer for the prepared SSPCM [23], as is shown in Figure 1c. The EG possessed a specific surface area of 32 m^2^/g, a micro thickness of 350 nm, and a particle size of 38 μm.

**Figure 2:**
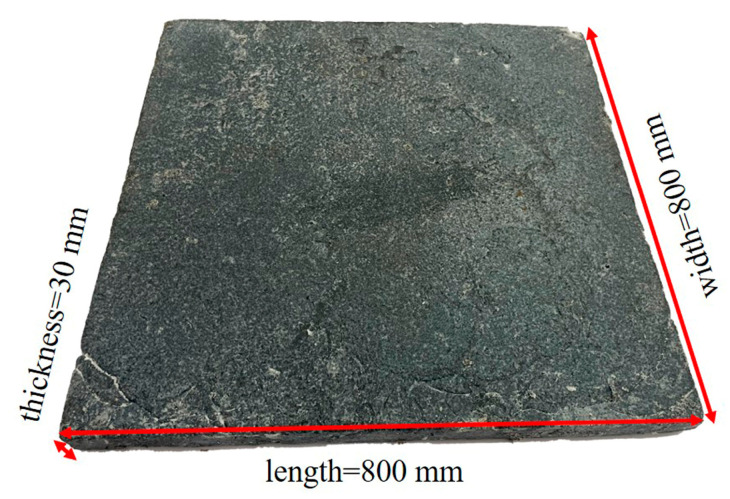
The prefabricated SSPCM plate.

The SSPCM was prepared by the melt blending method, as in our previous study [24]. The mass ratio of paraffin to HDPE was 8:2, and the mass of EG accounted for 2% of the total mass of paraffin and HDPE. First, paraffin was heated to 60 °C in an oil bath basin until it was melted completely. Second, the HDPE was mixed with the molten paraffin in an oil bath basin. The temperature of the oil was set to 160 °C with continuous stirring of 30 rpm for 30 min. The EG powder was then added into the oil bath basin and stirred at 30 rpm for 30 min. Finally, the mixture was poured into a rectangular mold and cooled to room temperature, forming the 800 × 800 × 30 mm SSPCM plate shown in Figure 2.

#### 2.1.2. The Properties of SSPCM

**Figure 3:**
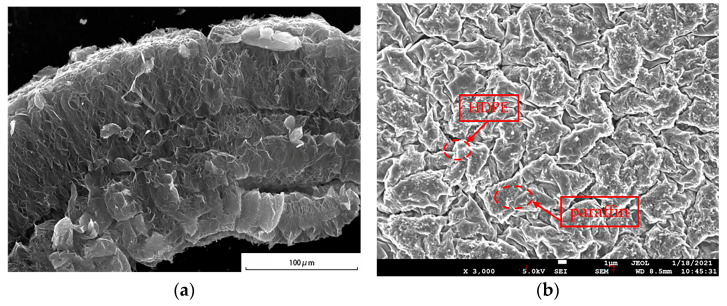
The SEM photographs of (**a**) EG and (**b**) the SSPCM.

The microstructure of the SSPCM was observed using a scanning electron microscope (SEM). The 10 × 10 × 3 mm SSPCM sample was dried and pre-processed by the metal-spraying process to enhance its conductivity [24]. Figure 3 displays the SEM of the EG and the SSPCM. As is shown in the Figure 3a, the EG presented a worm-like structure; the molten HDPE and paraffin can be absorbed well by the pores of the EG. In Figure 3b, the brighter part is the HDPE, while the darker parts are paraffin and EG [25]. The paraffin and EG were uniformly wrapped by the net structure of the HDPE, which proved that HDPE presented great compatibility with paraffin and EG [25].

**Figure 4:**
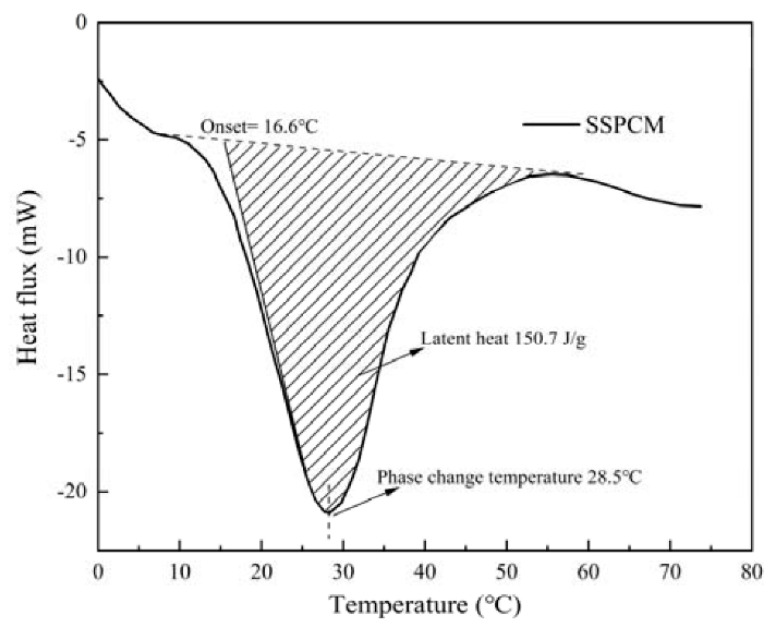
The DSC curve of the SSPCM.

A DSC-100 differential scanning calorimeter was used to measure the latent heat and phase change temperature of the SSPCM. The 10 mg SSPCM was placed into an aluminum crucible to be heated from room temperature to 150 °C at a heating rate of 10 °C/min. The development of heat flux with temperature was recorded automatically. As is shown in Figure 4, the latent heat of the SSPCM is 150.7 kJ/kg. The onset melting temperature and phase change temperature were 16.6 °C and 28.5 °C, respectively.

The thermal conductivities of the samples were measured using a DZDR-S Thermal Constants Analyzer. Two pieces of 100 × 100 × 30 mm SSPCM samples were set on the test plate, and the test probe was placed between the two SSPCM samples. The thermal conductivity of SSPCM was tested three times, and the values in the permissible error range of 3% were averaged as the final measurement value. The thermal conductivity of the SSPCM is 0.8 W/m·K, which is about four times higher than that of pure, solid paraffin.

The thermal stability of the SSPCM was measured using a high and low temperature-alternating damp heat test chamber with an accelerated thermal cycling analysis. An amount of 20 g of SSPCM was heated and cooled from room temperature (20 °C) to 80 °C during the 1000 cycles. The mass-loss ratio of PCM was only 0.9%, indicating the high thermal stability property of this SSPCM.

#### 2.1.3. The Establishment of the Thermal Experimental Platform

**Figure 5:**
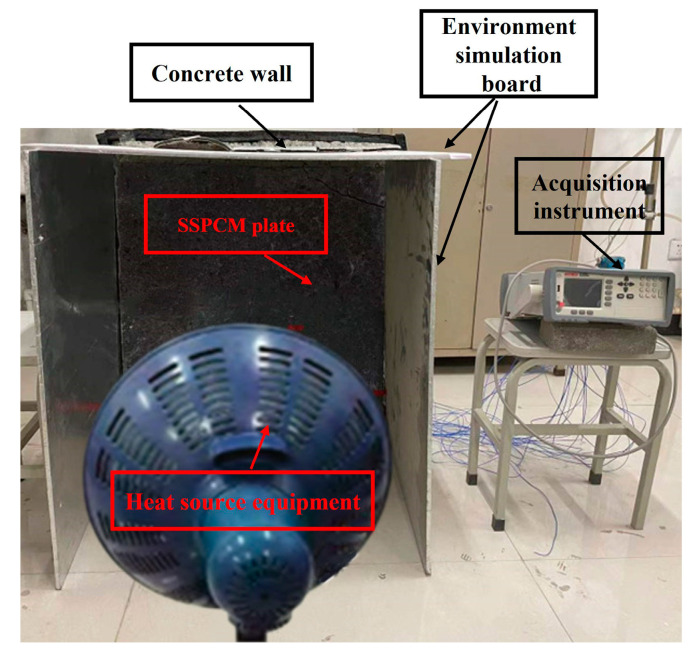
The experimental platform of the SSPCM granary wall.

An experimental platform representing the SSPCM granary wall was built, as is shown in Figure 5, which consisted of an SSPCM granary wall, a temperature-acquisition instrument (Agilent34970A), heat source equipment, and environment simulation boards. The SSPCM granary wall was constructed with a concrete layer of 800 × 800 × 210 mm and a prefabricated SSPCM plate of 800 × 800 × 30 mm. The four sides of the concrete layer were wrapped by 100 mm of insulated cotton. This ensured that the concrete layer was closed to an adiabatic boundary along its height and width. The heat source was mainly transferred from the thickness of the concrete layer. Moreover, the environment simulation boards were used to positively simulate the external temperature environment. During the experimental process, the heat source equipment was utilized to heat the surrounding air for two days. The external temperature environment was mainly regulated by adjusting the power of the heating source equipment and the distance from the SSPCM granary wall. The indoor and outdoor temperature data were effectively collected by the temperature-acquisition instrument in real time.

### 2.2. Numerical Section

#### 2.2.1. The Physical Model of the Granary Walls

**Figure 6:**
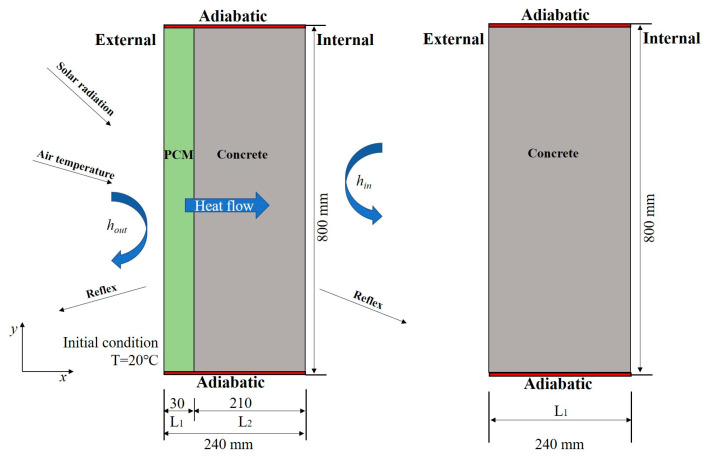
The heat transfer physical model of the granary walls.: (**left**) the SSPCM granary wall and (**right**) the common granary wall.

As is shown in Figure 6, the common granary wall consists of a 240 mm concrete layer. The SSPCM granary wall was formed by 30 mm of the SSPCM plate and 210 mm of the concrete layer, corresponding to its widths of L1 and L2, respectively. According to the DSC test, the phase change temperature of the SSPCM was 28.5 °C. The other parameters of SSPCM are listed in Table 1.

The ANSYS Fluent software was used to simulate the heat-transfer process of the SSPCM granary wall and the common granary wall. The internal and external boundaries of the granary wall were assumed to have convective heat transfer. Additionally, the top and bottom boundaries were set as adiabatic [28]. The initial temperature of the granary wall was 20 °C. According to the national standard of grain storage in China, the granary local temperature should be lower than 25 °C [13].

In order to preferably perform the heat transfer in the SSPCM granary wall, a structural, quadrilateral mesh with 4800 element numbers was employed to discretize the physical model of the SSPCM granary wall. The PISO (Pressure Implicit Split Operator) algorithm was used for coupling pressure and velocity. In the spatial discretization section, a least-squares cell-based evaluation was used for the gradient, and the second order was used for the pressure. The second order upwind was used for the momentum and energy equation. Furthermore, the first order implicit function was used for the transient formulation.

**Figure 7:**
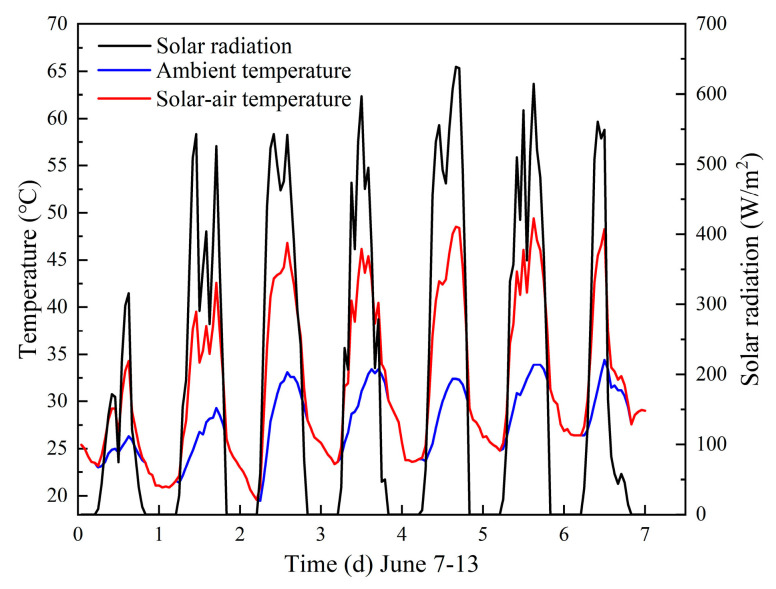
The solar–air temperature of Zhengzhou city [30].

Considering the thermal effect of solar radiation, a comprehensive solar–air temperature was adopted, shown in Figure 7, which was selected from a typical week in the summer in Zhengzhou city. The solar–air temperature (*T_sol_*_-*air*_) consists of solar radiation and ambient temperature (*T_air_*), as is shown in Equations (1) and (2).

(1) $$T_{sol-air}=T_{air}+\frac{\alpha I_{t}}{h_{out}}$$

(2) $$I_{t}=I_{D}+I_{d}+I_{Earth}$$

where *α* is the solar radiation absorption coefficient of the outdoor wall surface, 0.48; *h_out_* is the heat transfer coefficient of the outdoor wall surface, 18.3 W/m^2^·K [29]; *I_t_* is the total intensity of solar radiation; and W/m^2^ consists of the direct solar irradiance, *I_D_*, the diffuse horizontal irradiation, *I_d_*, and the ground-reflected irradiation *I_Earth_*, W/m^2^ [29].

#### 2.2.2. Mathematical Model

As was mentioned above, the SSPCM granary wall consisted of the SSPCM layer and the concrete layer. The bottom and top wall surfaces were under adiabatic conditions and the heat was applied in a uniform distribution along the y-direction. Here, a one-dimensional heat-transfer mathematical model along the x-direction was established for both the concrete and SSPCM layer; it is similar to those found in the literature [31,32,33]. The apparent heat capacity method was used to describe the latent heat transfer of the SSPCM.

For simplicity, the physical model of the SSPCM granary wall was established under the following assumptions:

(a) All layers of the wall system were assumed to be homogenous and isotropic;
(b) The heat transfer through the wall was one-dimensional;
(c) The thermal expansion of the materials was not considered;
(d) The thermophysical properties of the materials were constant, except for the change in the material properties of the SSPCM during the phase change interval;
(e) For multi-layered wall systems, the contact resistance between different layers was negligible.

The heat-transfer process nonlinearly changed with the time and ambient temperature. The heat-conduction equations of concrete and SSPCM can be expressed by Equations (3) and (4), respectively.

(3) $$\rho_{1}c_{1}\frac{\partial T}{\partial t}=\frac{\partial }{\partial x}(k_{1}\frac{\partial T}{\partial x})$$

(4) $$\rho_{SSPCM}c_{SSPCM}\frac{\partial T}{\partial t}=\frac{\partial }{\partial x}(k_{SSPCM}\frac{\partial T}{\partial x})$$

where $\rho_{1}$ and $\rho_{SSPCM}$ represent the densities of concrete and SSPCM, respectively, kg/m^3^; $c_{1}$ and $c_{SSPCM}$ represent the specific heat capacity of concrete and SSPCM, respectively, J/kg·K; and $k_{1}$ and $k_{SSPCM}$ represent the thermal conductivity of concrete and SSPCM, respectively, W/m·K. Temperature is represented by T, °C; and t is time, s.

Equations (5) and (6) are used to account for the change in material properties during the phase-transition process of SSPCM. The specific heat capacity is presented by Equation (7) for different phase-change states.

(5) $$\rho_{SSPCM}=\theta_{s}\rho_{s}+\theta_{l}\rho_{l}$$

(6) $$k_{SSPCM}=\theta_{s}k_{s}+\theta_{l}k_{l}$$

(7) $$c_{SSPCM}(T)=\left\{ \begin{array}{l} c_{SSPCM},T<T_{m}-\Delta T,\;T\geq T_{m}+\Delta T\\ \delta (T)\Delta H+c_{SSPCM},T_{m}-\Delta T\leq T<T_{m}+\Delta T \end{array}\right.$$

(8) $$\delta (T)=\frac{\exp(-{\left(T-T_{m}\right)}^{2})/{\left(\Delta T\right)}^{2})}{\Delta T\sqrt{\pi }}$$

where θ is the volume proportion of the SSPCM; $\theta_{s}$ and $\theta_{l}$ are the volume proportions of the solid phase and liquid phase in the SSPCM, respectively; $\rho_{s}$ and $\rho_{l}$ are the densities of solid SSPCM and liquid SSPCM, respectively, kg/m^3^; $k_{s}$ and $k_{l}$ are the thermal conductivity of solid SSPCM and liquid SSPCM, respectively, W/m·K; $c_{SSPCM}\left(T\right)$ is the equivalent specific heat capacity of SSPCM, which depends on temperature; $T_{m}$ is the phase-change temperature of SSPCM; 2Δ*T* is the range of phase-change temperature; and Δ*H* is the latent heat of SSPCM. $T_{m}$, Δ*T*, and Δ*H* can be obtained from the DSC test. The Gaussian function of the equivalent specific heat is represented by *δ*(*T*), which is expressed by Equation (8).

The boundary conditions of the external and internal wall surfaces can be described by Equations (9) and (10), respectively.

(9) $$-k_{SSPCM}\frac{\partial T}{\partial x}=h_{out}(T_{solar-air}-T)$$

(10) $$-k_{1}\frac{\partial T}{\partial x}=h_{in}(T_{in}-T)$$

where *h_in_* is the heat transfer coefficient of the internal surface, 8.7 W/m^2^·K [30]; and *T_in_* is the internal air temperature, °C.

Here, the adiabatic boundaries of the bottom and top wall surfaces can be formulated in Equations (11) and (12).

(11) $$\frac{\partial T}{\partial y}=0,\;y=0\;$$

(12) $$\frac{\partial T}{\partial y}=0,\;y=800\;$$

where *y* is the height direction of the SSPCM granary wall, in mm.

The initial condition is described in Equations (13) and (14).

(13) $$T=T_{0},0\leq x\leq 240\mathrm{mm},t=0$$

(14) $$T_{in}=T_{0},t=0$$

where *T*_0_ is the initial temperature, set at 20 °C.

#### 2.2.3. Thermal Performance Evaluation Indexes of the SSPCM Granary Wall

In order to quantitatively evaluate the heat-transfer performance and the temperature-regulation ability of the granary wall, some evaluation indexes [34,35,36] were used, including the temperatures of the internal wall surface and the SSPCM layer, heat flux, and energy saving.

(1) The temperature of the internal wall surface and the SSPCM layer

The external heat energy was gradually transferred to the internal wall surface and the SSPCM layer. The greater the decrease in temperature in the internal wall surface, the better the energy storage efficiency of the SSPCM was. The phase change process can be observed from the temperature development of the SSPCM layer.

(2) Heat flux on the internal surface of the wall

This is also known as the heat flux (*ϕ*) on the inner surface of the wall, referring to the heat energy passing through the inner surface per unit of time. The smaller the heat flux, the less heat transfers to the inner surface.

(15) $$\phi =-kA\frac{dT}{dx}$$

(3) Energy saving rate

The cooling load is the total amount of reduced heat through the inner surface of the wall within one week, 168 h. The energy saving value is expressed by the ratio of the difference in cooling load between the common granary wall and the SSPCM granary wall to the common granary wall [12]. The greater the ratio, the greater the energy saving was.

(16) $$Q=\int_{0}^{168}\phi dt$$

(17) $$E_{saving}=\frac{Q_{0}-Q_{PCM}}{Q_{0}}$$

where *Q* is the cooling load, W·h/m^2^; *Q*_0_ is the cooling load of the common granary wall, W·h/m^2^; *Q_PCM_* is the cooling load of the SSPCM granary wall, W·h/m^2^; and *E_saving_* is the energy saving rate, %.

### 2.3. The Experiment Result and Model Validation

**Figure 8:**
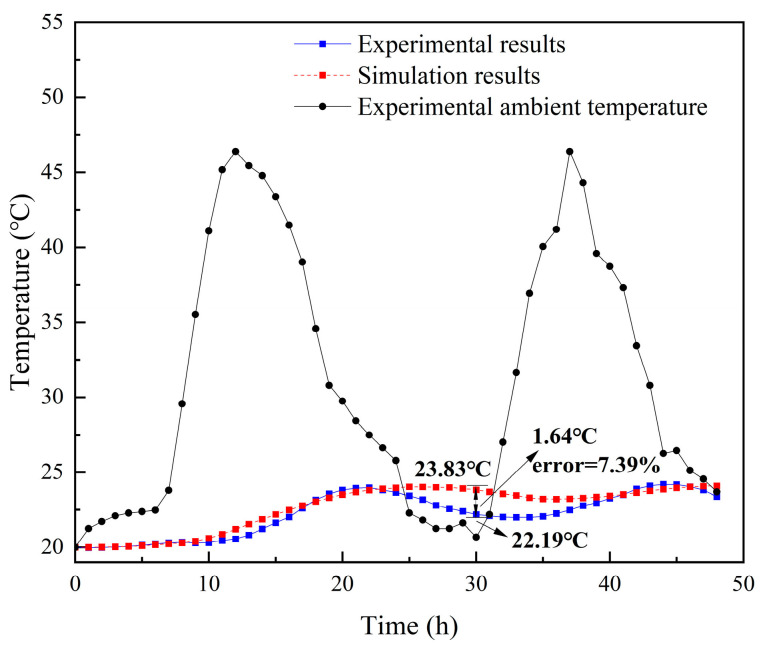
The comparison between the experiment and calculated results.

To validate the reasonability of the physical model, the experimental results were compared with the simulation results. As is shown in Figure 8, the calculated results of the internal surface temperatures on the SSPCM granary wall were close to the experimental values. The internal surface temperature varied with the variation of the external ambient temperature. For a comparison at 30 h, the indoor surface temperature of the simulation was, at maximum, 1.64 °C higher than the temperature measured in the experiment. The maximum relative error was approximately 7.39%. The relative errors were mainly caused by the permissible error of ±0.8 °C in the accuracy of the temperature-acquisition instrument and the neglected contact thermal resistance between the SSPCM plate and the concrete wall.

## 3. Results and Discussion

**Figure 9:**
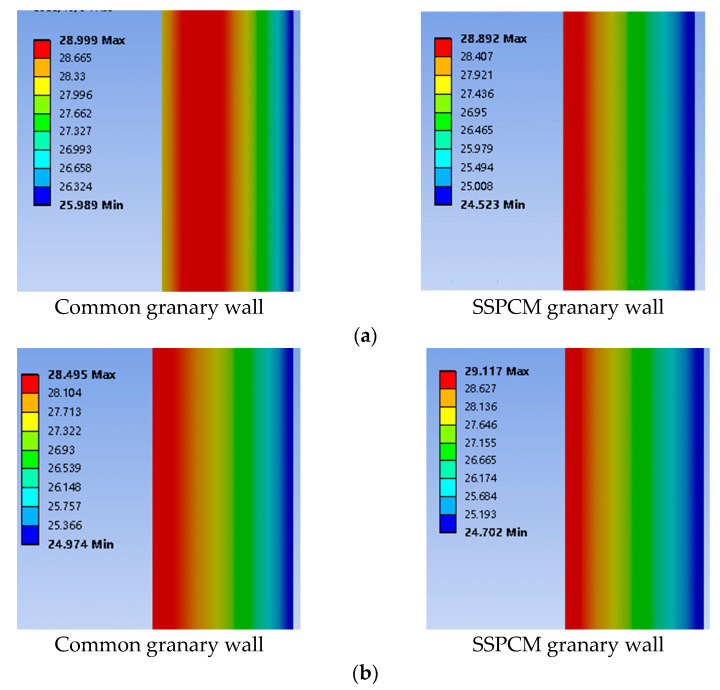
The temperature distribution of the common and SSPCM granary walls at (**a**) T = 72 h (third day) and (**b**) T = 168 h (seventh day).

In order to optimize the design parameters of the granary, the heat-transfer performance of SSPCM granary walls was investigated by comparing it to that of the common granary walls. Figure 9 shows the results of the temperature contour between the common and SSPCM granary walls. Here, the SSPCM layer was 30 mm thick with a latent heat of 150 kJ/kg, a phase-transition temperature of 28.5 °C, and a thermal conductivity of 0.8 W/m·K.

As can be seen from Figure 9a, on the third day, the red margin in the SSPCM granary wall was smaller than the red margin in the common granary wall, showing a lower temperature and a narrower distribution range in the red region. This is because the latent heat of the SSPCM can effectively absorb the external heat energy to decrease the amount of heat transfer into the indoor space. Further, the thermal conductivity of the SSPCM was lower than that of concrete; therefore, the heat resistance and heat inertia of the SSPCM granary wall was higher than that of the common granary wall. Thus, less heat was conducted to the SSPCM granary wall when compared to the common granary wall. The SSPCM granary wall demonstrated a superior temperature-regulation performance.

In Figure 9b, at the end of the 7th day, the red high-temperature region and the internal surface temperature of the common granary wall were still higher than that of the SSPCM granary wall, although the distribution range gradually became smaller. Moreover, the maximum external surface temperature of the common granary was lower than that of the SSPCM granary wall, due to the fact that concrete can quickly release heat energy. Summarily, the SSPCM plays a key role in self-regulating the temperature of the SSPCM granary wall to greatly decrease the granary energy consumption. Thus, it may provide green grain storage without extra energy consumption, which meets the requirement of green grain storage.

### 3.1. The Effect of the SSPCM Thickness on the Heat Transfer

It is necessary to optimize the thickness of the phase change material for maximum use and economic efficiency. To compare the effect of the SSPCM thickness on the heat-transfer performance of granary walls, four SSPCM granary-wall models were simulated in this paper. These were named SSPCM-0, SSPCM-10, SSPCM-20, SSPCM-30, and SSPCM-40. SSPCM-0 represented the common granary wall without the SSPCM plate. SSPCM-10, SSPCM-20, SSPCM-30, and SSPCM-40 represented the SSPCM granary wall with 10 mm, 20 mm, 30 mm, and 40 mm SSPCM layers, respectively.

**Figure 10:**
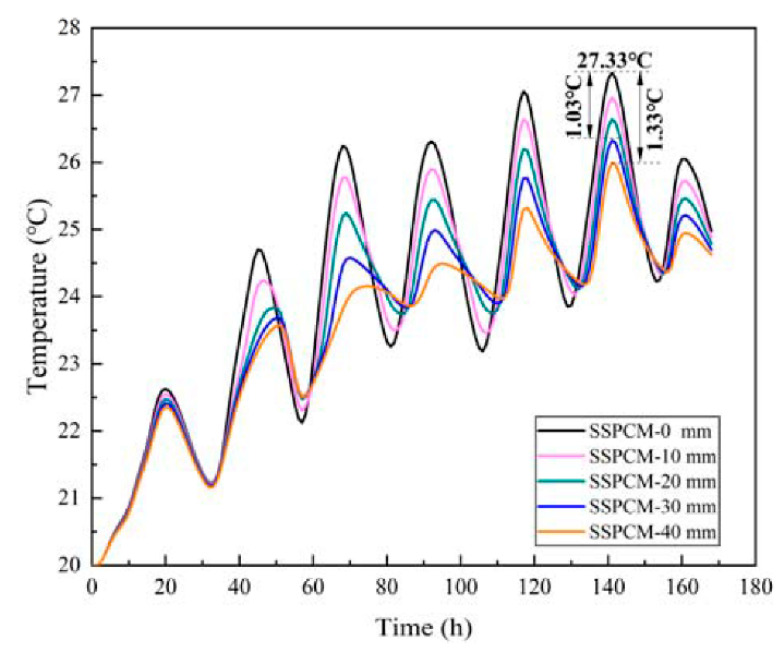
The internal surface temperature of SSPCM granary wall with different SSPCM layer thicknesses.

Figure 10 displays the temperature variations of the internal wall surface with different SSPCM layer thicknesses. The temperature of the internal wall surface decreased with the increasing thickness of the SSPCM layer. On the sixth day, the maximum temperature of the internal wall surface was 27.33 °C for SSPCM-0 without any SSPCM. Additionally, the temperatures of SSPCM-30 and SSPCM-40 were 26.3 °C and 26 °C, which were 1.03 °C and 1.33 °C lower than that of SSPCM-0, respectively. The SSPCM layer can effectively decrease the internal surface temperature of the SSPCM granary wall.

**Figure 11:**
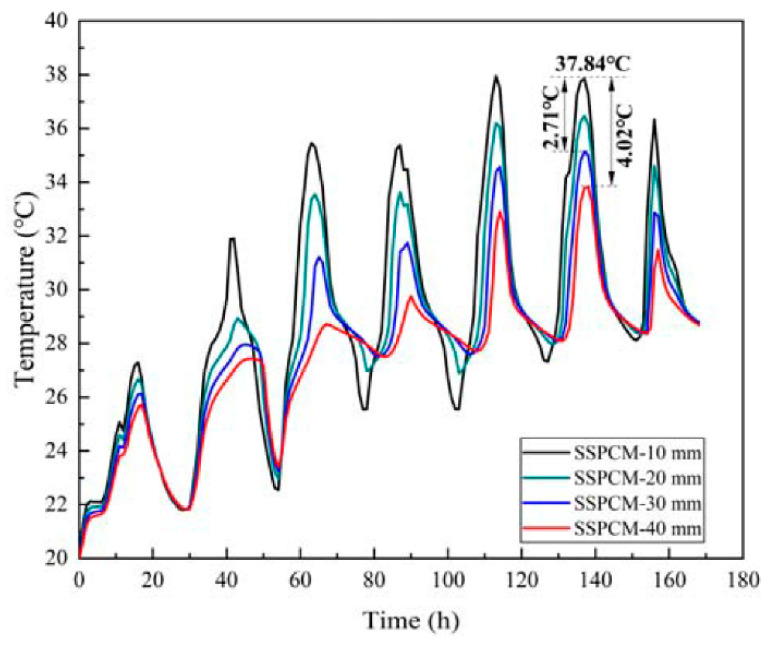
The internal surface temperature of the SSPCM layer with different SSPCM layer thicknesses.

The peak temperature in the SSPCM layer also showed a similar trend to that of the internal wall surface, as is shown in Figure 11. On the sixth day, the SSPCM layers had completed their phase transition with a total utilization of their latent heat. The peak temperatures of the SSPCM layer surfaces were 37.84 °C, 36.47 °C, 35.13 °C, and 33.82 °C, corresponding to SSPCM-10, SSPCM-20, SSPCM-30, and SSPCM-40, respectively. In comparison to the SSPCM-10, the internal surface temperature of SSPCM-30 and SSPCM-40 were reduced by 2.71 °C and 4.02 °C, respectively. With the increasing increment of SSPCM thickness, a higher external heat was absorbed in the SSPCM layer.

**Figure 12:**
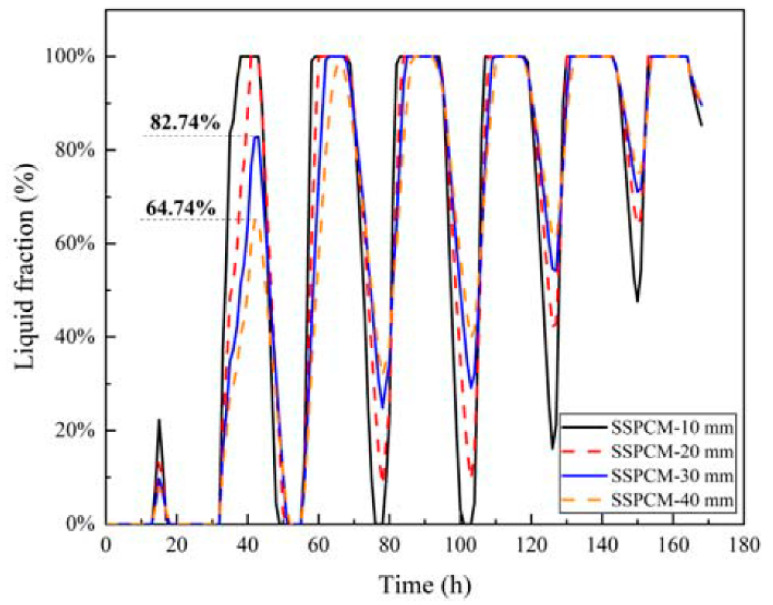
The liquid fraction of the SSPCM layer with different SSPCM thicknesses.

Figure 12 displayed the liquid fraction of four kinds of SSPCM layers. The liquid fraction of the SSPCM layer gradually increased with the rise of external temperature. During the 32 h to 50 h period, the maximum liquid fraction of SSPCM-30 and SSPCM-40 was 82.73% and 64.74%, respectively. Both values then completely recovered to the solid state. However, during the 120 h to 140 h period, all kinds of SSPCM layers were quickly and completely changed from a solid state to a liquid state. Furthermore, none could entirely recover to a solid state due to the continuously high ambient temperature. Thus, based on the reduction in the internal surface peak temperature of the SSPCM granary wall and the SSPCM layer and the liquid fraction of the SSPCM layer, the SSPCM plate with a 30 mm thickness is optimal.

### 3.2. The Effects of the Prefabricated SSPCM Plate Location on the Heat Transfer

**Figure 13:**
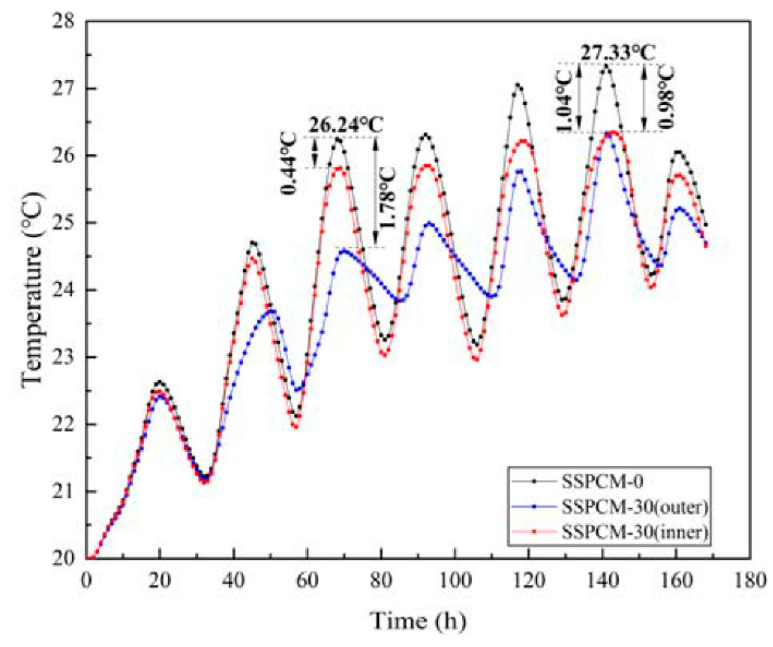
The internal surface temperature of SSPCM granary wall with different position.

Figure 13 displays the temperature variations of the internal wall surface when the SSPCM layer was at different locations on the granary wall. Here, the SSPCM thickness is 30 mm, and the thermal conductivity of the SSPCM is 0.8 W/m·K. The SSPCM plate was installed on the outer and inner sides, respectively.

As can be seen from Figure 13, the SSPCM layer location indeed affected the heat transfer of the wall. During the 65 h to 72 h period, the peak internal surface temperature of the common granary wall was 26.24 °C. When compared with the common granary wall, the maximum temperatures of the SSPCM granary wall surface with inner and outer locations were 25.88 °C and 24.46 °C, corresponding to the temperature differences of 0.44 °C and 1.78 °C, respectively. However, in the two cases of the SSPCM wall, the temperature was still lower than that of the common granary wall. During the 140 h to 150 h period, the maximum internal surface temperatures were 27.33 °C, 26.29 °C, and 26.35 °C, corresponding to the common granary wall, SSPCM granary wall with the SSPCM layer installed on the outer location, and inner location, respectively. The temperatures of the SSPCM granary walls were reduced by 1.04 °C and 0.98 °C when compared to the common granary wall. It was indicated that the SSPCM installed on the external side of the granary wall could provide a lower temperature for the granary wall in Zhengzhou city. Furthermore, the internal wall surface was near the grain storage requirement of 25 °C, which could save more energy in achieving the requirement [13].

**Figure 14:**
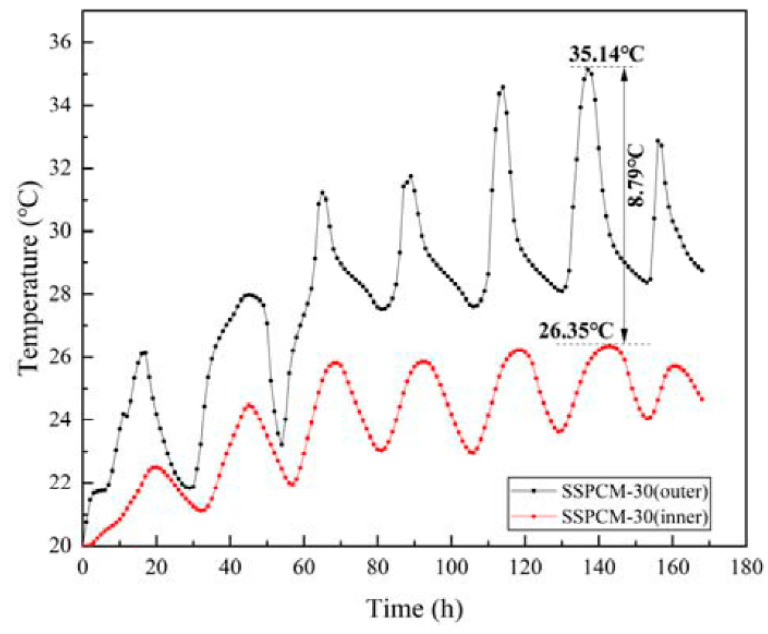
The internal surface temperature of the SSPCM layer at different SSPCM locations.

Figure 14 represents the internal surface temperature distribution of the SSPCM layer at different SSPCM positions. When installed on the outer side, the SSPCM layer had a higher internal surface temperature than the SSPCM layer located on the internal side. During the 135 h to 145 h period, the maximum temperature difference was 8.79 °C between the external and internal SSPCM layer locations. The temperature difference between the SSPCM layer locations was mainly caused by the thermal insulation effect of the concrete layer. When the SSPCM layer was installed on the external side of the granary wall, the solar radiation and ambient temperature directly affected the SSPCM layer. The heat energy was primarily absorbed by the SSPCM latent heat and was then gradually transferred to the internal surface. However, when the SSPCM layer was installed on the internal side of the granary wall, the solar–air temperature was greatly decreased by the concrete layer. In this case, its temperature variation was slower than that of the SSPCM layer installed on the external side.

In summary, when the SSPCM layer is applied to the external granary wall, the internal surface temperature is lower than that of the inner wall. In Zhengzhou city, an SSPCM plate located on an external wall may provide better temperature regulation for a PCM granary wall.

### 3.3. The Effects of the Thermal Conductivity

Thermal conductivity has a controversial effect on the temperature regulation of the walls. It is well known that decreasing thermal conductivity will prevent more heat flux from being conducted into the indoor space [37]. However, with respect to the SSPCM plate with a large volume, enhancing the thermal conductivity can improve the phase change rate and the utilization of the whole phase change latent capacity (improving the liquid ratio), whereby the SSPCM plate will restrain more heat from entering indoors [33]. Therefore, the comprehensive effects of thermal conductivity need to be considered emphatically.

**Figure 15:**
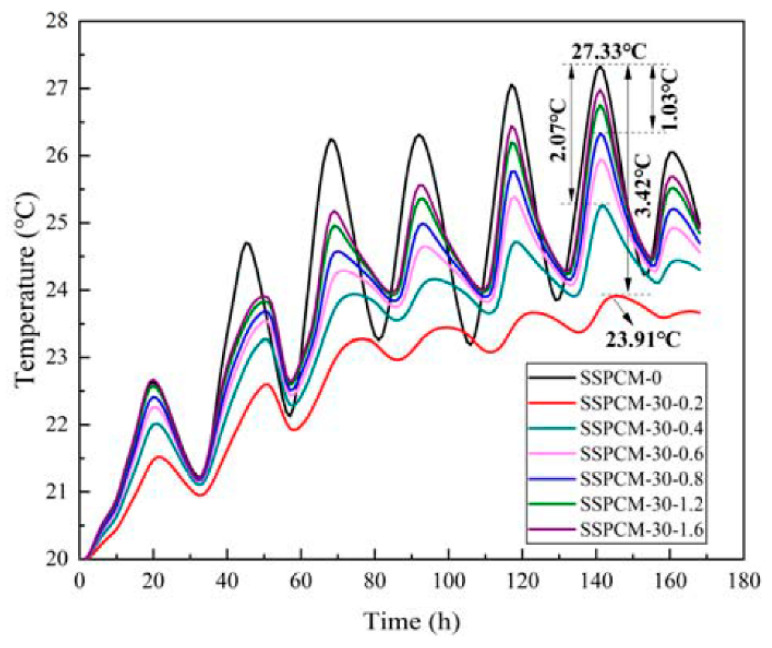
The internal surface temperatures of the SSPCM granary wall with different thermal conductivities.

The internal surface temperature of SSPCM at different thermal conductivity conditions is displayed in Figure 15. In these cases, the SSPCM thickness was 30 mm and the SSPCM layer was located on the outer wall. The thermal conductivity of SSPCM was set as 0.2, 0.4, 0.6, 0.8, 1.2, and 1.6 W/m·K, respectively.

**Figure 16:**
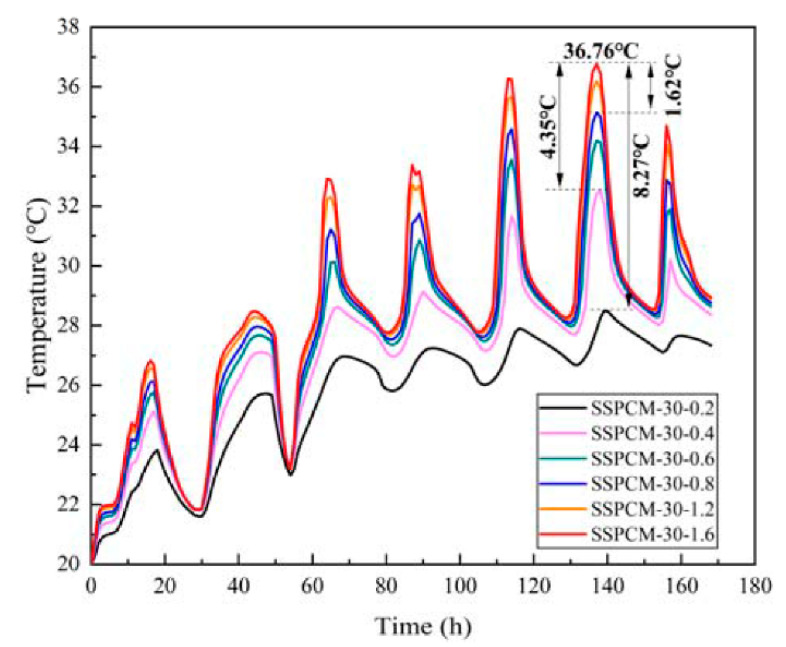
The internal surface temperature of the SSPCM layer at different thermal conductivities.

As can be seen from Figure 15, the internal surface temperature decreased with the decreasing of the thermal conductivity of SSPCM. Under the same ambient conditions, the lower the thermal conductivity, the lower the internal surface temperature of the wall was. When the thermal conductivity was 0.2 W/m·K, the internal surface temperature of the wall was the minimum 23.91 °C, which was 3.42 °C lower than when the thermal conductivity was 1.6 W/m·K. This was followed by 2.07 °C and 1.03 °C differences when the thermal conductivities of SSPCM layers were 0.4 W/m·K and 0.8 W/m·K, respectively. This phenomenon can be also proven using the temperature revolution of the SSPCM plate at different thermal conductivities, as is shown in Figure 16. The maximum peak temperature difference of the SSPCM layer was 8.27 °C between the thermal conductivities of 0.2 W/m·K and 1.6 W/m·K. This indicates that lowering the thermal conductivity did indeed decrease the heat conduction flux into the walls.

**Figure 17:**
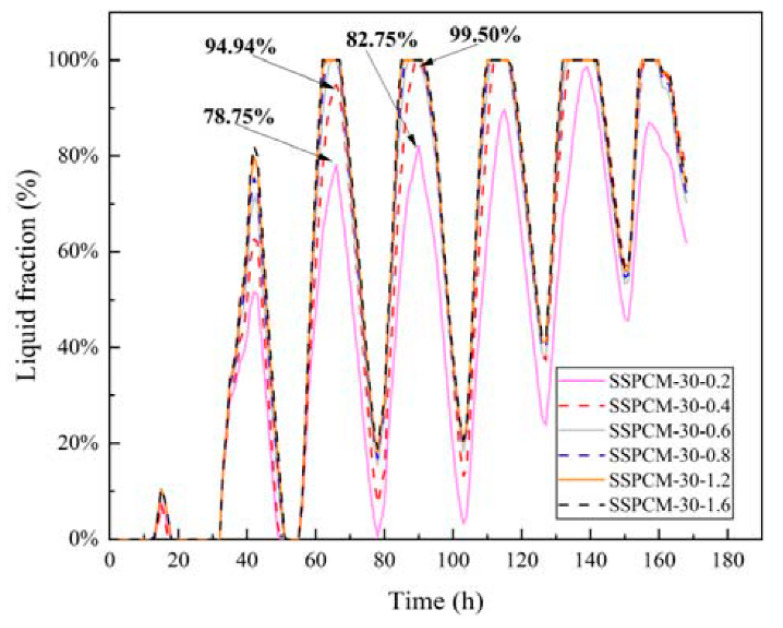
The liquid fraction of the SSPCM layer with different thermal conductivities.

However, lowering the thermal conductivity decreased the liquid fraction and the utilization of the phase-change latent capacity. Figure 17 shows the liquid fractions of the SSPCM layers at different thermal conductivities. During the 55 h to 100 h period, the SSPCM layers completely changed to a liquid state when the thermal conductivity of the SSPCM layer was higher than 0.4 W/m·K. For example, the peak liquid fraction of SSPCM layers was 78.75% and 82.75% at 72 h and 96 h, respectively, for a thermal conductivity of the SSPCM plates of 0.2 W/m·K. However, for the case of a thermal conductivity of 0.4 W/m·K, the peak liquid fractions of the SSPCM layers were 94.94% and 99.50% at the same time. This indicates that enhancing the thermal conductivity did indeed increase the utilization of the phase-change latent capacity.

Together, compared with the fact that enhancing thermal conductivity can increase the utilization rate of the latent capacity, the heat resistance caused by low thermal conduction plays a major role in controlling the temperature rise of the internal wall. This is due to the fact that the SSPCM was incorporated in the form of flat plate whose surface direction uniformly received the ambient heat. While the thickness magnitude (30 mm) was quite small compared to the direction of the plane (800 mm), the phase change resistance along the thickness direction was relatively low, leading to little demand on the thermal conductivity enhancement.

Furthermore, the effect of the thermal conductivity of the SSPCM on the energy-saving effect is investigated in this section. As is detailed in Table 2, the energy-saving rate with the effect of SSPCM being incorporated into the granary wall can be obtained by Equation (14). The cooling load (Q) of the common granary wall and the SSPCM granary wall at different thermal conductivities was 5996.43, 3848.16, 4729.11, 5139.63, 5375.09, 5634.28, and 5774.07 W·h/m^2^, respectively. When compared to the common granary wall, the energy-saving rates of the SSPCM granary wall were 35.83%, 20.13%, 14.29%, 10.36%, 6.04%, and 3.71%. The energy-saving effect was gradually improved with a decrease in the thermal conductivity of the SSPCM layers. When the SSPCM layer was installed on the external side of the SSPCM granary wall, the SSPCM layer not only used the latent heat to store the heat energy, but also resisted the heat-energy transition to the indoor environment. The lower thermal conductivity can resist more heat energy transfer, which mainly caused the downtrend of the energy-saving effect with the increase of thermal conductivity in the SSPCM layer.

Summarily, in these cases, the optimal parameters of the granary wall are: a 30 mm thick SSPCM plate installed on the outer location of the granary wall. A smaller thermal conductivity is recommended. In this paper, with all the optimal parameters used in the granary walls, the requirements for quasi-low-temperature grain storage can be satisfied.

## 4. Conclusions

In this paper, the prefabricated SSPCM plate was used to construct a SSPCM granary wall. When compared with the common granary wall, the heat transfer characteristics of the SSPCM granary wall were simulated to optimize its thermal regulation performance for green grain storage. The main conclusions are as follows:

(1) The SSPCM granary wall was prefabricated with the self-temperature-regulated behavior of the PCMs. The optimal parameters of the SSPCM may meet the great demand for green grain storage for large-capacity granaries in China;
(2) Increasing the thickness of the SSPCM layer can reduce the temperature and heat flux of the internal wall surface to achieve the energy-saving effect. In Zhengzhou city, the SSPCM layer located on the outer wall surface can enhance the temperature-regulation effect of the SSPCM granary wall;
(3) With the increasing thickness of the SSPCM, the internal surface temperature decreased. However, in comparison to the maximum thickness of 40 mm, both the maximum latent heat utilization rate and the energy-storage effect are obtained when the SSPCM layer thickness is 30 mm;
(4) Thermal conductivity has a controversial effect on the temperature regulation of the walls. A smaller thermal conductivity dominates in the thermal regulation of walls by restraining the heat flux conducted to the indoors. The maximum energy-saving rate was up to 35.83% for the outer SSPCM layer with a thickness of 30 mm and a thermal conductivity of 0.2 W/m·K. The optimal utilization of the SSPCM layer was present when the thermal conductivity was 0.4 W/m·K.

## Figures and Tables

**Table 1:** Material properties of concrete and SSPCM [26,27].

Material	Density (kg/m^3^)	Specific Heat Capacity (J/kg·K)	Phase Change Temperature (°C)	Latent Heat (J/g)	Thermal Conductivity (W/m·K)	PCM Thickness (mm)
Concrete	2400	1030	N/A	N/A	1.74	N/A
SSPCM	790	1500	28.5	150	0.8	10, 20, 30, 40
					0.2, 0.4, 0.6, 0.8, 1.2, 1.6	30

**Table 2:** Cooling load and energy saving rate for the PCM granary wall.

Parameters	Common Granary Wall	PCM Granary Wall Thermal Conductivity of PCM (W/m·K)					
		0.2	0.4	0.6	0.8	1.2	1.6
*Q* (W·h·m^−2^)	5996.43	3848.16	4729.11	5139.63	5375.09	5634.28	5774.07
Δ*Q* (W·h·m^−2^)	/	2148.27	1267.32	856.8	621.34	326.15	222.36
*E_saving_* (%)	/	35.83	21.13	14.29	10.36	6.04	3.71

## Data Availability

Not applicable.
